# The Importance of Motherhood for Early Maternal Bonding and Engagement

**DOI:** 10.1007/s10995-026-04272-1

**Published:** 2026-05-20

**Authors:** Karina M. Shreffler, Kayleigh Esparza, Tara Wyatt

**Affiliations:** 1 Fran and Earl Ziegler College of Nursing, University of Oklahoma Health Campus, 1100 N. Stonewall Ave., Oklahoma City, OK 73117 USA; 2 Laureate Institute for Brain Research, Tulsa, OK USA; 3 Department of Human Development and Family Science, Oklahoma State University, Stillwater, OK USA

**Keywords:** Importance of motherhood, Bonding, Maternal, Pregnancy, Postpartum

## Abstract

**Introduction:**

Maternal perceptions about the importance of motherhood have been found to impact a variety of maternal well-being outcomes, but the impact of the importance of motherhood for the early maternal bonding relationship and on caregiving behaviors has not been explored. Examining attitudinal factors associated with maternal-infant bonding or early caregiving behaviors is essential for the development of effective interventions.

**Methods:**

Using a clinic-based, urban sample of predominately low-income and diverse pregnant women (*N* = 177), we used hierarchical linear regression analysis to examine whether perceptions of the importance of motherhood measured during the first trimester predicted maternal-fetal bonding measured during the second trimester, caregiving engagement at two months postpartum, and postpartum bonding measured at six months postpartum, controlling for sociodemographic characteristics and prior outcomes.

**Results:**

Perceived importance of motherhood was positively and significantly associated with higher maternal-fetal bonding (*b* = 1.28, *p* < .01), greater daily infant engagement (*b* = 0.57, *p* < .05), and higher postpartum bonding (*b* = 0.94, *p* < .01). Adjusting for prior outcomes to model temporal pathways revealed that the impact of perceived importance of motherhood for early engagement was fully attenuated by maternal-fetal bonding but remained a significant predictor of postpartum bonding.

**Discussion:**

Perceiving motherhood as important promotes early maternal bonding and caregiving behaviors, which are critical for early infant development. Preconception or parenting education programs that strive to promote the importance of motherhood through mindfulness or maternal reflective functioning interventions should be considered.

## Significance

What is Already Known on this Subject?

What this Study Adds?

Perceived importance of motherhood is associated with various maternal outcomes, including childbearing intentions and distress following involuntary childlessness and pregnancy loss.

Perceptions of the importance of motherhood in early pregnancy are associated with early maternal bonding and caregiving behaviors. Screening for and enhancing women’s attitudes about motherhood and becoming a mother might be a critical target for intervention.

## Introduction

Motherhood is a salient identity for the majority of women in the U.S. (Katz-Wise et al., 2010). On average, women desire to have two children, and fewer than 15% of women report intentions to remain childless (Guzzo & Hayford, 2023). Perceived importance of motherhood has been associated with a variety of maternal outcomes, such as childbearing intentions (Fletcher-Hilderbrand, et al., 2022; McQuillan et al., 2015), plans to adopt (Park & Hill, 2014), distress about childlessness (McKennon Brody & Frey, 2017; Inhorn et al. 2017; McQuillan et al., 2012) and distress about pregnancy loss (Erato et al. 2021; Shreffler et al., 2011). There is wide variation in how important women rate motherhood to be (McQuillan et al., 2008; Tichenor et al., 2017), and not all women have positive expectations for motherhood (Copeland and Harbough 2019). When mothers are unable to form positive expectations about motherhood during pregnancy, they are at risk of bonding disturbance (Rusanen et al., 2021), but it remains unclear how perceiving motherhood as important is associated with the early maternal-infant bonding relationship or caregiving behaviors.

Because the early bonding relationship and caregiving engagement is critical for infant development (e.g., Alhusen et al., 2013; Le Bas et al., 2020; Rocha et al., 2020), it is critical to identify modifiable targets for early intervention. Attitudes are a common precursor to relevant behaviors (Ajzen et al., 2018) and are more easily modified through educational and behavioral interventions than other factors such as personality (Conner & Norman, 2005). Perceived importance of motherhood refers to attitudes about the meaning and value of being a mother and is not necessarily a stable characteristic of women; perceptions of the importance of motherhood differ by life course circumstances including having children and experiencing infertility (McQuillan et al., 2008), and they can change over time following experiences such as pregnancy loss (Erato et al. 2021). Additionally, maternal awareness of the role and motherhood expectations have been shown to be modifiable through mindfulness-based intervention (Passaquindici et al., 2024; Sajadian et al. 2022).

In this study, we used longitudinal data to examine how women’s perceptions of the importance of motherhood in early pregnancy were associated with maternal-fetal bonding during the second trimester, daily infant engagement at two months postpartum, and bonding at six months postpartum.

## Methods

### Sample

Data for the current study came from a longitudinal, urban, clinic-based cohort study involving 177 pregnant women (age range = 16–38; 89% receiving public insurance) recruited in 2017–2018 from their first prenatal appointment. The university-affiliated participating clinics serve a racially diverse and economically under-resourced patient population. The sample for the current study was restricted to the 116 participants who responded to survey assessments from the first trimester of pregnancy through six months postpartum. Most attrition occurred between the first and second waves of the study, coinciding with the first and second trimesters of pregnancy, with the primary known reason for attrition due to pregnancy loss. Response rates are comparable to other longitudinal clinical studies with a diverse, economically disadvantaged sample (Nicholson et al., 2015). The study was approved by the [university masked for review] Institutional Review Board.

### Procedures

This study includes four time points, with recruitment occurring at the first prenatal visit (average gestation age of 10 weeks) and spanning into the sixth month postpartum. Informed consent/assent with parental consent for participants under 18 was performed at the time of enrollment. Participants received $50 for participation in the first assessment, which included blood and saliva draws, and $40 for participation in subsequent online survey assessments. This study uses measures collected prenatally in the first and second trimesters, and postnatally at two and six months postpartum.

### Measures

The *Importance of motherhood* scale assessed the feelings and attitudes women had about motherhood in early pregnancy. The 4-item (“I think my life will be or is more fulfilling with children”; “I always thought I would be a parent”; “Having children is important to my feeling complete as a woman”; and “It is important for me to have children”) scale was developed by McQuillan et al. (2008) and was measured in the current study during the first trimester. Each item was rated on a 5-point scale ranging from 1 (*Strongly disagree*) to 5 (*Strongly agree*). The 4 items were summed (α = 0.74 in the current sample) to create the importance of motherhood scale score (*range* = 4–20).

The *Prenatal Attachment Inventory* was used to assess maternal-fetal bonding during the second trimester. The 21-item (e.g., “I feel love for the baby”; “I try to imagine what the baby is doing in there”) scale was developed by Müller and Mercer (1993). Each item was rated on a 4-point scale ranging from 1 (*Almost never*) to 4 (*Almost always*). The 21 items were summed in the current sample (α = 0.93) to create the final mother-fetal bonding score (*range* = 21–84).

*Daily engagement in caregiving activities* (e.g., activities such as feeding, diapering, bathing, and reading to the baby) was measured at two months postpartum using the Fragile Families Caregiving Scale (McLanahan & Garfinkel, 2000). The scale measures how many times per week these caregiving activities were performed, with scores ranging from 0 (item was performed 0 days a week) to 7 (item was performed 7 days a week). The responses for the eight items were summed to obtain a score of daily engagement across the week (*range* = 0–56).

*Postpartum bonding* was measured at the six-month postpartum assessment. This 24-item Postpartum Bonding Questionnaire was developed by Brockington et al. (2006) and was administered at six months postpartum. Each item (e.g., “I enjoy playing with my baby”; “My baby is the most beautiful baby in the world”) was rated on a 6-point scale ranging from 1 (Always) to 6 (Never). The 24 items were reverse scored and summed in the current sample (α = 0.88) to create the final postpartum bonding score (*range* = 24–144) such that higher scores indicate higher maternal-infant bonding.

Participants provided demographic information at enrollment. Several variables potentially associated with early bonding and caregiving engagement are included in this study. Maternal *age* is included as a continuous variable. Due to little variation in income among the sample, an indicator of *economic hardship* was used that represents whether the respondent experienced six hardships in the past year due to a shortage of money such as being homeless, unable to pay rent or mortgage, or went without meals. Participants answered yes (1) or no (0) regarding whether or not they experienced financial hardships in the past year, and responses were summed to create an index variable (*range* = 0–6). Union status was coded such that those who were *married/cohabiting* were coded as 1 and all others coded as 0. *Parity* was included as a continuous variable of the number of children prior to the current pregnancy the respondent has given birth to (*range* = 0–9). Whether or not a pregnancy was coded as *unintended* was based on two survey questions (0 = pregnancy was reported to have been planned or that the participant had been trying to become pregnant; 1 = pregnant was reported to be unplanned or that the participant was trying to avoid pregnancy when it occurred). *Race/ethnicity* was measured using standard Census questions about race and Hispanic ethnicity. Variables were coded into Black, Native American, and Hispanic dummy variables, with non-Hispanic White as the reference category.

### Analytic Plan

Descriptive statistics were first computed, followed by a temporal sequence of linear regression analyses in which earlier outcomes predicted later outcomes while adjusting for importance of motherhood scores and sociodemographic covariates. The first analysis examines the association between importance of motherhood measured in the first trimester and maternal-fetal bonding measured in the second trimester, controlling for sociodemographic characteristics. The second analysis examines the association between importance of motherhood scores and daily infant engagement measured at two months postpartum, adjusting for sociodemographic variables. A second model adds maternal-fetal bonding as a predictor of daily infant engagement. The third analysis examines the association between importance of motherhood scores and postpartum bonding measured at six months postpartum, adjusting for sociodemographic variables. A second model adds predictors of maternal-fetal bonding and daily engagement to capture temporal pathways.

## Results

### Descriptive Statistics

Descriptive statistics on study variables and demographics are presented in Table 1. Maternal-fetal bonding scores (*M* = 63.65; *SD* = 12.40; *Range* = 36–84) were similar to prior studies using the PAI to assess prenatal bonding among women in the second trimester (e.g., Busonera et al., 2017; Coflkun et al., 2019; Gürsoy et al. 2025). On average, participants reported fairly high levels of daily engagement with their infants (*M* = 43.09; *SD* = 7.87; *Range* = 10–56) measured at two months postpartum and maternal-infant bonding (*M* = 122.10; *SD* = 10.27; *Range* = 58–129) measured at six months postpartum. Similar to US-based national data on perceived importance of motherhood suggesting high importance, particularly among mothers (e.g., McQuillan et al., 2008), importance of motherhood scores among study participants were high, on average (*M* = 16.11; *SD* = 3.07; *Range* = 4–20).

On average, participants were 25.86 (*SD* = 5.54) years old and had experienced more than one (*M* = 1.53; *SD* = 1.89) economic hardship in the past year. More than half (62%) reported being married or living in a cohabiting union, and on average, participants had more than one child prior to the focal pregnancy (*M* = 1.31; *SD* = 1.38). Approximately 40% of the sample reported identifying as White, 28% as Black, 13% as Hispanic, and 18% as Native American. The racial/ethnic breakdown of the sample is similar to demographics reported in the 2020 Census for the urban area where data collection occurred (March of Dimes, 2026; NICWA 2022).

**Table 1:** Descriptive statistics of study variables (*N* = 125)

Variables	M or %	SD	Range
Maternal-fetal bonding^a^	63.65	12.40	36–84
Daily infant engagement^b^	43.09	7.87	10–56
Maternal-infant bonding^c^	122.10	10.27	58–129
Importance of motherhood	16.11	3.07	4–20
Sociodemographic variables			
Age	25.86	5.54	16–38
Economic hardship	1.53	1.89	0–6
Union	62%		0–1
Parity	1.31	1.38	0–9
Unintended pregnancy	52%		0–1
Race/ethnicity			
White	52%		0–1
Black	32%		0–1
Hispanic	13%		0–1
Native	21%		0–1

^a^measured in 2nd trimester; ^b^measured at 2 months PP; ^c^measured at 6 months PP. All other variables measured in the 1 st trimester

### Linear Regression Analyses

As shown in Table 2, in the models adjusting only for sociodemographic characteristics, higher levels of perceived importance of motherhood scores were associated with greater maternal-fetal bonding in the second trimester (*b* = 1.28; *p*<.01), greater daily infant engagement at two months postpartum (*b*=0.57; *p*<.05), and higher maternal-infant bonding at six months postpartum (*b*=0.94; *p*<.01). However, after additionally adjusting for maternal-fetal bonding, the association between importance of motherhood and early infant engagement was attenuated and no longer significant, whereas maternal-fetal bonding was a significant predictor of early infant engagement (*b*=0.16; *p*<.05). In the analysis examining maternal-infant bonding outcomes, importance of motherhood scores were attenuated but remained significant (*b*=0.67; *p*<.05) after adding maternal-fetal bonding and daily infant engagement predictors to the model. Additionally, early infant engagement predicted maternal-infant bonding at six months postpartum (*b*=0.43; *p*<.001).

**Table 2:** Linear regression of bonding and parenting behaviors by importance of motherhood and sociodemographic characteristics (*N* = 125)

	Maternal-fetal bonding (2nd tri)			Daily infant engagement (2 mo PP)						Maternal-infant bonding (6 mo PP)					
	M1			M1			M2			M1			M2		
Variables	b		SE	b		SE	b		SE	b		SE	b		SE
Importance of motherhood	1.28	**	0.36	0.57	*	0.24	0.37		0.25	0.94	**	0.29	0.67	*	0.29
Maternal-fetal bonding							0.16	*	0.06				0.03		0.08
Daily infant engagement													0.43	***	0.12
Sociodemographic variables															
Age	− 0.42		0.24	0.15		0.16	0.19		0.16	− 0.22		0.19	− 0.26		0.19
Economic hardship	0.27		0.60	− 0.09		0.41	− 0.13		0.41	−1.02	*	0.48	− 0.99	*	0.48
Union	−4.29		2.41	−1.74		1.63	− 0.97		1.63	−4.53	*	1.93	−3.76		1.90
Parity	− 0.42		0.79	−1.23	*	0.52	−1.12	*	0.52	−1.35	*	0.62	− 0.82		0.62
Unintended pregnancy	−6.32	**	2.25	−1.76		1.52	− 0.62		1.56	−5.22	**	1.79	−4.62	*	1.82
Race/ethnicity															
White (ref)															
Black	−3.01		2.87	−3.50		1.94	−3.08		1.94	−1.64		2.28	− 0.70		2.28
Hispanic	0.94		3.70	0.42		2.42	0.75		2.43	2.20		2.91	1.59		2.83
Native	−1.91		3.14	1.07		2.12	1.48		2.09	2.10		2.51	1.32		2.44
Constant	60.91	***	9.32	34.66	***	6.26	25.02	**	7.32	121.14	***	7.43	104.68	***	8.99
Adj. R^2^	0.16			0.07			0.12			0.22			0.31		

***p<.001; **p<.01; *p<.05

## Discussion

This is the first study, to our knowledge, to examine how maternal perceptions about the importance of motherhood in early pregnancy impact the development of the early maternal-infant relationship. This study suggests that attitudes or values about motherhood that exist during pregnancy can shape both bonding and parenting behavior outcomes. Perceptions about the importance of motherhood in the first trimester predicted maternal-fetal bonding during the second trimester, daily infant engagement at two months postpartum, and maternal-infant bonding at six months postpartum. Additionally, results suggested evidence of temporal pathways; perceived importance of motherhood predicted maternal-fetal bonding, which predicted later daily infant engagement, which in turn predicted postpartum maternal-infant bonding. As bonding and early caregiving behaviors are critical for child developmental outcomes (e.g., Alhusen et al., 2013; Le Bas et al., 2020; Rocha et al., 2020), these findings point to a potential area of intervention in early pregnancy: perceived importance of motherhood.

Attitudes and values are often precursors of later behaviors (Ajzen et al., 2018), and they are changeable (Conner & Norman, 2005; Maio & Haddock, 2007). Perceived importance of motherhood is not a static attitude but rather can change due to experiences or reflection (Erato et al. 2021; Passaquindici et al., 2024; Sajadian et al. 2022). Changes in attitudes about motherhood have been found to occur due to childbirth experiences (Ruble et al., 1990), transition to parenthood (Baxter et al., 2015), employment during motherhood (Borrell-Porta et al., 2023), and the experience of a pregnancy loss (Erato et al. 2021). In order to intervene to change attitudes, it is critical to understand the content, structure, and function of attitudes (Maio & Haddock, 2007). Understanding the contextual influences, experiences, and social norms that impact perceived importance of motherhood might be useful for the design of interventions focused on promoting the early maternal-infant relationship.

### Strengths, Limitations, Future Directions

This study has several key strengths. First, the longitudinal design allows for examination of the development of the early bonding relationship between pregnancy to six months postpartum. The longitudinal nature of the sample also enables an investigation into how perceived importance of motherhood during early pregnancy predicts maternal outcomes later in pregnancy and into the postpartum period. Second, the sample is comprised of predominately low-income and racially diverse participants, adding valuable insights to the literature on the importance of motherhood and the early maternal-child relationship for underrepresented and understudied populations. Interestingly, although prior studies using US-based data from 2004 to 2007 have noted differences in importance of motherhood scores by race/ethnicity and socioeconomic status (e.g., McQuillan et al., 2008; Tichenor et al., 2017), we did not find significant differences at the bivariate level (not shown), and sample averages in key variables of interest were similar to those reported in other studies. It is unclear whether group differences reported in data that are nearly two decades old no longer exist or whether there might be local cultural factors that reduce differences seen in other samples. Moreover, this study used validated scales to measure the various outcomes of interest, which ensures greater reliability and accuracy of the findings. Finally, this study provides unique insights on how perceptions about the importance of motherhood in early pregnancy matter for early bonding and caregiving outcomes.

There are several limitations as well that should be addressed in future research. Due to the clinical nature and single site of the sample, the generalizability of study findings is unclear. There is also the possibility of unmeasured antecedents and confounders that may influence perceptions of the importance of motherhood prior to conception and the development of the maternal-child relationship after birth. Future research should utilize a larger, nationally representative longitudinal sample to increase generalizability; survey assessments of participants before, during, and after pregnancy would enable a study on the development of attitudes and perceptions about the importance of motherhood and their impact on maternal-child bonding and caregiving behaviors. Future research exploring potential group differences in perceptions about motherhood and whether those are differently associated with maternal and child outcomes would also be useful to improve measurement for diverse samples.

### Practical Implications

Screening for and enhancing women’s attitudes about motherhood and becoming a mother might be a critical target for intervention. Preconception education about motherhood and the transition to parenthood might help shape women’s perceptions about the importance of motherhood as they consider pregnancy or recognize a pregnancy. The development and implementation of interventions to support new mothers should begin prenatally and continue into the postpartum period (Commission on Social Determinants of Health, 2008; Marmot, 2010). Targeted interventions such as parenting education programs that focus on parental reflective functioning, for example, can play a crucial role in strengthening maternal-child bonding though maternal reflections about the importance of motherhood for bonding and developmental outcomes (Huynh et al., 2024). Perinatal healthcare providers and childbirth education instructors should consider discussing the meaning of motherhood, particularly with those about to become mothers for the first time, before or during pregnancy (Shreffler et al., 2019; Zdolska-Wawrzkiewicz et al., 2019). During prenatal visits, practitioners might consider offering ultrasounds and allowing mothers to listen to the heartbeat of the fetus to enhance prenatal bonding (de Jong-Pleij et al., 2013; Ji et al., 2005; Nishikawa & Sakakibara 2013; Sedgmen et al. 2006; Shreffler et al., 2019). Furthermore, mothers should be informed during these visits about community-based resources that provide emotional and instrumental support to new mothers, which might help to reduce stressors and improve bonding (Ciciolla et al. 2024; Kay 2024). In summary, identifying ways to enhance the maternal-infant relationship as early as possible is key for the promotion of maternal and child well-being.

## Data Availability

The data are available from the corresponding author upon request.
